# Genomic characterization of a novel sakobuvirus (family *Picornaviridae*) from a European badger (*Meles meles*) in Hungary

**DOI:** 10.1007/s00705-025-06234-4

**Published:** 2025-02-20

**Authors:** Péter Pankovics, Benigna Balázs, Ákos Boros, Gábor Nagy, Sándor Szekeres, Gábor Reuter

**Affiliations:** 1 Department of Medical Microbiology and Immunology, Medical School, University of Pécs, Pécs, Hungary; 2 Department of Animal Physiology and Health, Hungarian University of Agriculture and Life Science, Kaposvár Campus, Kaposvár, Hungary; 3 Department of Parasitology and Zoology, University of Veterinary Medicine, Budapest, Hungary; 4 HUN-REN-UVMB Climate Change: New Blood-Sucking Parasites and Vector-Borne Pathogens Research Group, Budapest, Hungary; 5 Department of Medical Microbiology and Immunology, University of Pécs, Szigeti út 12, Pécs, H-7624 Hungary

## Abstract

Since 2012, sakobuvirus and sakobuvirus-like (family *Picornaviridae*) sequences have been detected in felines, wild boars, badgers, and fur seals. In this study, we determined the genome sequence of a novel sakobuvirus identified in faecal samples from European badgers (*Meles meles*) in Hungary. The strain SakV/badger/B40B/2022/HUN (PQ382029) has a type IV internal ribosome entry site (IRES) and'barbell-like' structures in its 5' and 3’UTRs, with a genomic layout that matches the L-1AB-1C-1D/2A^H − Box/NC^-2B-2C/3A-3B-3C-3D structure. The most closely related strain, SaKoV/Badger/3A_2019/ITA (OP293080), shows 82.8%, 82.5%, and 80.4% amino acid sequence identity in the proteins P1, 2C, and 3CD, respectively, indicating that different sakobuviruses can potentially circulate among members of the same host species in geographically different regions.

**Supplementary Information:**

The online version contains supplementary material available at 10.1007/s00705-025-06234-4.

The family *Picornaviridae* encompasses a broad range of viruses [1], and within this diverse family, the genus *Sakobuvirus* is a relatively recent discovery. Sakobuviruses have received increasing attention due to their expanding host range and geographic presence. *Sakobuvirus aportufeli* is the only officially recognized species in the genus. The first member of this species was identified in faeces of a domestic cat in Portugal in 2012 [2], after which closely related sequences were found in faecal samples from wild boar (*Sus scrofa*) in China [3], and cats in Italy [4]. Interestingly, potential novel sakobuvirus (SakV) sequences have also been identified in the faeces of subantarctic and South American fur seals (*Arctocephalus* spp.) [5] and European badgers (*Meles meles*) [6]. These discoveries provide growing evidence that SakVs are more widespread in wildlife than previously thought, reinforcing the need for further research into the host range and diversity of the members of this genus.

Hence, we report the identification and complete genome characterization of a novel SakV obtained from a European badger (*Meles meles*) in Hungary. A total of 13 faecal and 12 tissue specimens from European badgers were investigated [7]. Sakobuvirus-like reads were identified using viral metagenomic and next-generation sequencing, while the full genome sequence was determined through RT-PCR amplification and Sanger sequencing (Supplementary Methods). The genome sequence of the strain SakV/badger/B40B/2022/HUN has been deposited in the GenBank database under accession number PQ382029.

A total of 10 sakobuvirus-like reads were identified through metagenomic sequencing, eight of which had 51–76% amino acid (aa) sequence identity to sakobuvirus A isolate FFUP1/Portugal/2012 (YP_008802671) and were verified by RT-PCR (Supplementary Fig. S1). At that time, no sakobuvirus (SakV) sequences from badgers were available in the GenBank database, but since then, in 2022, the genome sequence of a SakV isolate from a badger in Italy was reported [6], and this was included in our further analysis. The genome of the European badger SakV strain from the current study, SakV/badger/B40B/2022/HUN (PQ382029), is 8,168 nt in length (Fig. 1). Its 5’ untranslated region (UTR) is 693 nt in length and contains a type IV internal ribosome entry site (IRES) with 67.8%, 67.5%, and 84.2% nt sequence identity to those of feline (KF387721), wild boar (MW660837), and badger (OP293080) SakV isolates, respectively (Supplementary Figs. S2 and S4A). The 3'UTR is 212 nt in length and exhibits 49.2%, 36.2%, and 63.8% nt sequence identity to feline, wild boar, and badger SakVs, respectively, and a predicted “barbell-like” secondary structure with primary loop and poly(Y) regions could be identified (Supplementary Fig. S3 and S4B). The start codon (AUG) is located at nt position 694–696, and the complete coding sequence is 7,263 nt long, encoding a 2,420-aa polyprotein (Fig. 1). The strain from this study showed 73.8%/78% nt/aa sequence identity to SaKoV/Badger/3A_2019/ITA (OP293080) and 61%/58.7% nt/aa sequence identity to FFUP1/Portugal/2012 (NC_022802) strains. This strain contains unique amino acid cleavage sites at the VP1/2A (Y/T) and 2B/2C (H/A) junctions, while the cleavage sites at the L/VP0 (Q/G) and 3B/3C (Q/S) junctions are identical to those found in badger SakV, and the VP0/VP3 (P/Q) site aligns with that of feline SakV. The cleavage sites VP3/VP1 (Q/A), 2A/2B (Q/G), 2C/3A (Q/G), 3A/3B (Q/G), and 3C/3D (Q/S) are identical across the three species (Fig. 1A). Conserved aa motifs that were identified previously in feline [2] and badger [6] SakVs were also found in the isolate from this study. The P1/2C^hel^/3C^pro^3D^pol^ proteins showed 82.8%, 82.5%, and 80.4% identity to the corresponding regions of strain SaKoV/Badger/3A_2019/ITA, and 60.7%, 66.3%, and 63.5% identity, respectively, to those of strain FFUP1/Portugal/2012 (Fig. 1B). No evidence of recombination was detected among the three sakobuvirus genomes compared, and strain SaKoV/Badger/3A_2019/ITA was found to be the closest relative, exhibiting the highest nucleotide sequence identity across all genomic regions (Fig. 1B). Phylogenetic analysis based on the P1/2C^hel^/3C^pro^3D^pol^ proteins, performed by the maximum-likelihood method, showed that the strain from this study clusters with SaKoV/Badger/3A-2019/ITA (OP293080) on a branch alongside feline (KF387721) and wild boar (MW660837) SakVs, corresponding to the species *Sakobuvirus aportufeli* (Fig. 2). Although the Italian [6] and Hungarian badger SakV strains might together represent a new SakV species, they are likely to be regarded as separate genotypes. No additional SakV sequences were found in the collected samples.

The family *Picornaviridae* encompasses a wide variety of viruses [8, 9]. Within this family, members of the genus *Sakobuvirus* are most closely related to human saliviruses (genus *Salivirus*) and kobuviruses (genus *Kobuvirus*). These viruses share significant genetic similarity and infect a variety of host species. Since 2012, SakVs have been found in Europe (Portugal and Italy) [2, 4, 6], South America (Brazil) [5], and East Asia (China) [3] and have been detected in felines, fur seals, wild boars, and badgers [2–6]. SakVs are likely to constitute at least two distinct species. Viral sequences from felines and wild boars belong to the species *Sakobuvirus aportufeli*, while the isolates from European badgers in Italy and Hungary appear to belong to a separate species. Although there is no formal subgrouping within these species, differences in amino acid sequences between the badger SakV strains suggest the existence of two distinct types, comparable to the subtype variations that have been described within the species *Kobuvirus aichi*. Sakobuvirus-like sequences have also been detected in fur seals [5], but these sequences exhibit substantial divergence from both the feline and badger SakV strains. Unfortunately, the fur seal sequences provide only partial genome coverage, and the analysis in this study therefore relied exclusively on BLASTp-based comparisons (Supplementary Fig. S5). The isolate from this study clusters closely with previous isolates from European badgers, suggesting a strong association between specific virus strains and their host species. However, further investigations, including virus isolation and experimental infections, are required to better understand the pathogenic potential of this virus, its associated clinical manifestations, and host immune responses. The detection of SakVs in both terrestrial and aquatic predators highlights their broad ecological adaptability and potential for widespread distribution across diverse environments. This underscores the necessity of further research to investigate the transmission dynamics and evolutionary patterns of SakV, and their potential implications for both animal and human health.

**Fig. 1:**
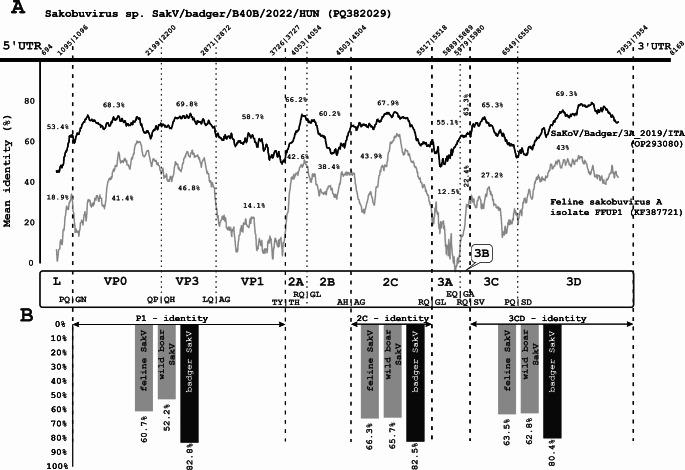
Comparative analysis of the novel badger sakobuvirus (BaSakV) strain SakV/badger/B40B/2022/HUN (PQ382029) and members of the genus *Sakobuvirus*. (**A**) Genome structure of the study strain and its mean nucleotide identity values in comparison to the corresponding regions of the reference strain FFUP1/Portugal/2012 (KF387721) of the species *Sakobuvirus aportufeli*. The figure was generated using SimPlot software, using the the complete coding nucleotide sequences of three sakobuviruses, with the following settings: window size, 400 bp; step size, 5 bp; GapStrip set to "Off"; and the F84 ("Maximum Likelihood") model with a transition/transversion ratio of 2.0. The amino acid (aa) cleavage sites were predicted based on comparisons to the available sakobuvirus sequences. (**B**) Percent identity of the P1, 2C, and 3CD aa sequences of the study strain to the corresponding regions of (1) feline sakobuvirus A isolate FFUP1 (KF387721) (species *Sakobuvirus aportufeli*), (2) wild boar sakobuvirus isolate WBSA (MW660837), and (3) the badger sakobuvirus strain SaKoV/Badger/3A_2019/ITA (OP293080). The values were obtained using the Sequence Identity And Similarity (SIAS) web service (http://imed.med.ucm.es/Tools/sias.html) with default settings and the "Length of Multiple Sequence Alignment" options

**Fig. 2:**
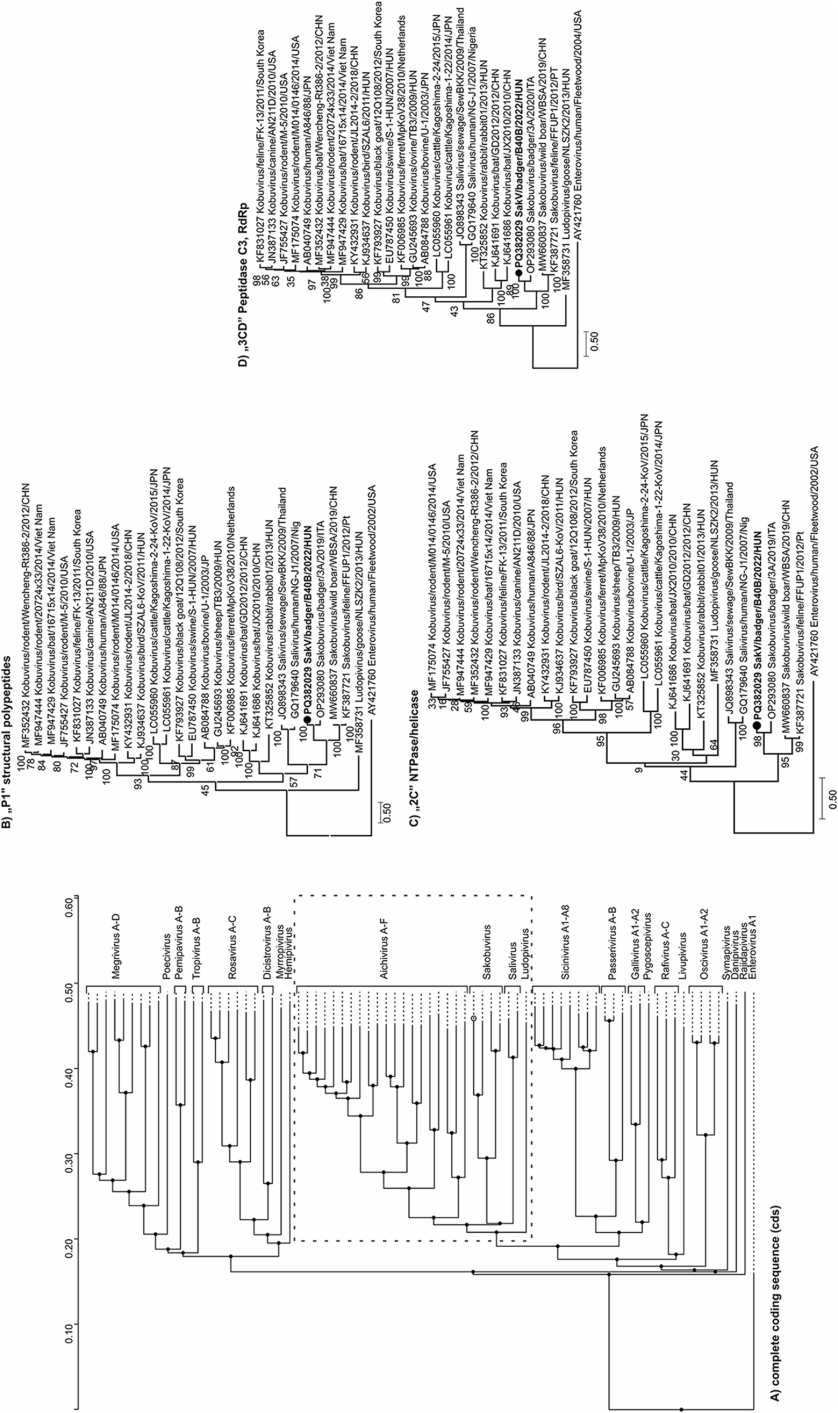
Phylogenetic analysis of badger sakobuvirus strain SaKoV/Badger/B40B/2022/HUN (PQ382029) (bold letter) and representative members of the family *Picornaviridae*. (**A**) Phylogenetic relationship of 22 representative members of the subfamily *Kodimesavirinae* based on amino acid comparisons of the complete coding sequences. (**B-D**) Phylogenetic analysis based on the amino acid sequences of the P1 (**B**), 2C (**C**), and 3CD (**D**) regions, which are demarcated by a dashed line (Supplementary Table S1). The study strain and the previous badger isolate belong to a phylogenetic lineage that is separate from isolate FFUP1 (KF387721)

## Electronic Supplementary Material

Below is the link to the electronic supplementary material

Supplementary Material 1

Supplementary Material 2

Supplementary Material 3

Supplementary Material 4

Supplementary Material 5

Supplementary Material 6

Supplementary Material 7

Supplementary Material 8

Supplementary Material 9
