# Supplementary material for: Genomic characterization of a novel sakobuvirus (family Picornaviridae) from a European badger (Meles meles) in Hungary
Source: Arch Virol. 2025 Feb 20;170(3):63. doi: 10.1007/s00705-025-06234-4 (PMC11842475; doi:10.1007/s00705-025-06234-4)
Supplement: Supplementary file 3 — Supplementary Material 3 [file 705_2025_6234_MOESM3_ESM.docx]

**Genomic characterization of a novel sakobuvirus (family *Picornaviridae*) from a European badger (*Meles meles*) in Hungary**

Supplementary file

**Supplementary Table S1.: The names and identification numbers of the sequences used for the phylogenetic analysis from the subfamily *Kodimesavirinae* are in alphabetical order according to their current taxonomic name.**

| Genus | Species | Virus name | Isolate | Accession |
| --- | --- | --- | --- | --- |
| Danipivirus | Danipivirus abrafi | danipivirus A1 | IDEXX/ZfPV-1/2017/USA | MH368041 |
| Dicipivirus | Dicipivirus acadici | cadicivirus A1 | 209 | JN819202 |
|  | Dicipivirus berinro | cadicivirus B1 | hedgehog/H14/2015/HUN | MF188967 |
| Gallivirus | Gallivirus afowli | gallivirus A1 | turkey/M176/2011/HUN | JQ691613 |
|  |  | gallivirus A2 | 518C | KF979337 |
| Hemipivirus | Hemipivirus aholtge | hemipivirus A1 | LPXYC213122 | MG606089 |
| Kobuvirus | Kobuvirus aichi | aichivirus A1 | A846/88 | AB040749 |
|  |  | aichivirus A2 | dog-AN211D-USA-2009 | JN387133 |
|  |  | aichivirus A3 | USA/2010/M-5 | JF755427 |
|  |  | aichivirus A4 | FK-13 | KF831027 |
|  |  | aichivirus A5 | SZAL6-KoV/2011/HUN | KJ934637 |
|  |  | aichivirus A6 | 20724x33 | MF947444 |
|  |  | aichivirus A7 | RtMruf-PicoV/JL2014-2 | KY432931 |
|  |  | aichivirus A8 | MKV1/NYC/2014 | MF175074 |
|  |  | aichivirus A9 | Wencheng-Rt386-2 | MF352432 |
|  |  | aichivirus A10 | 16715x14 | MF947429 |
|  | Kobuvirus bejaponia | aichivirus B1 | U-1 | AB084788 |
|  |  | aichivirus B2 | MpKoV38/Netherlands/2010 | KF006985 |
|  |  | aichivirus B3 | sheep/TB3/HUN/2009 | GU245693 |
|  | Kobuvirus cebes | aichivirus C1 | swine/S-1-HUN/2007/Hungary | EU787450 |
|  |  | aichivirus C2 | 12Q108/South Korea/2012 | KF793927 |
|  | Kobuvirus dekago | aichivirus D1 | cattle/Kagoshima-1-22-KoV/2014/JPN | LC055961 |
|  |  | aichivirus D2 | cattle/Kagoshima-1-24-KoV/2015/JPN | LC055960 |
|  | Kobuvirus ecuni | aichivirus E1 | Rabbit01/2013/HUN | KT325852 |
|  | Kobuvirus femyomini | aichivirus F1 | BtMr-PicoV/JX2010 | KJ641686 |
|  |  | aichivirus F2 | BtMf-PicoV-2/GD2012 | KJ641691 |
| Livupivirus | Livupivirus asmonewi | livupivirus A1 | newt/II-5-Pilis/2014/HUN | KX463670 |
| Ludopivirus | Ludopivirus agrewhifrogo | ludopivirus A1 | goose/NLSZK2/HUN/2013 | MF358731 |
| Megrivirus | Megrivirus chigalli | megrivirus A1CP-CPOL | 2993D | HM751199 |
|  |  | megrivirus C1 | chicken/B21-CHV/2012/HUN | KF961186 |
|  |  | megrivirus C2 | 27C | KF979336 |
|  | Megrivirus aturhepa | megrivirus A2 | LY | KC663628 |
|  |  | megrivirus A3 | W18 | KY369299 |
|  | Megrivirus berockdo | megrivirus B1 | HK21 | KC876003 |
|  |  | megrivirus B2 | pigeon/GALII5-PiMev/2011/HUN | KC811837 |
|  | Megrivirus deharri | megrivirus D1 | harrier/MR-01/HUN/2014 | KY488458 |
|  | Megrivirus epengu | megrivirus E1 | penguin/KGI-BHS-P5/2015 | MF405436 |
| Myrropivirus | Myrropivirus achiwas | myrropivirus A1 | LPSF20501 | MG600081 |
| Oscivirus | Oscivirus ahokorobi | oscivirus A1 | 10717 | GU182408 |
|  |  |  | 007167 | GU182409 |
|  |  | oscivirus A2 | 10878 | GU182410 |
|  |  |  | 00742 | GU182411 |
| Passerivirus | Passerivirus ahokothi | passerivirus A1 | thrush/Hong Kong/00356/2007 | GU182406 |
|  |  |  | 00805 | GU182407 |
|  | Passerivirus bewaxi | passerivirus B1 | waxbill/DB01/HUN/2014 | MF977321 |
| Pemapivirus | Pemapivirus achisotu | pemapivirus A1 | WHWGC151314 | MG600106 |
|  | Pemapivirus brohepotu | pemapivirus B1 | WHWGC151314 | MG600108 |
| Poecivirus | Poecivirus ablacachi | poecivirus A1 | BCCH-449 | KU977108 |
| Pygoscepivirus | Pygoscepivirus apingu | pygoscepivirus A1 | 991 | MH255796 |
| Rafivirus | Rafivirus atorti | rafivirus A1 | UF4 | KJ415177 |
|  | Rafivirus begecki | rafivirus B1 | PXYC222841 | MG600090 |
|  | Rafivirus crhima | rafivirus C1 | cane toad/AU1/Australia/2017 | MG967619 |
| Rajidapivirus | Rajidapivirus ashaska | rajidapivirus A1 | DHBYCGS18742 | MG600093 |
| Rosavirus | Rosavirus acaliforni | rosavirus A1 | mouse/USA/M-7/2010 | JF973686 |
|  |  | rosavirus A2 | hu/GA7403/Gambia/2008 | KJ158169 |
|  | Rosavirus brorati | rosavirus B1 | RNCW0602091R | KX783423 |
|  | Rosavirus chewhibe | rosavirus C1 | RASK8F | KX783424 |
|  |  | rosavirus C2 | RATLC11A | KX783425 |
|  |  | rosavirus C3 | NCGX12IN | KX783432 |
| Sakobuvirus | Sakobuvirus aportufeli | sakobuvirus A | FFUP1/Portugal/2012 | KF387721 |
|  |  |  | WBSA | MW660837 |
|  | unassigned | unassigned | SaKoV/Badger/3A_2019/ITA | OP293080 |
| Salivirus | Salivirus aklasse | salivirus A1 | hu/NG-J1/Nigeria/2007 | GQ179640 |
|  |  | salivirus A2 | hu/FHB/China/2011 | KM023140 |
| Sicinivirus | Sicinivirus ahiberni | sicinivirus A1 | chicken/UCC001/Eire | KF741227 |
|  |  | sicinivirus A2 | ch/JSY/China/2014 | KP779642 |
|  |  | sicinivirus A3 | ch/RS/BR/2015/2 | KY069113 |
|  |  | sicinivirus A4 | ch/100C/Hong Kong/2009 | KF979332 |
|  |  | sicinivirus A5 | ch/RS/BR/2015/1 | KY069112 |
|  |  | sicinivirus A6 | ch/RS/BR/2015/4R | MG846481 |
|  |  | sicinivirus A7 | ch/RS/BR/2015/7S | MG846484 |
|  |  | sicinivirus A8 | ch/RS/BR/2015/3R | MG846482 |
| Symapivirus | Symapivirus atriliza | symapivirus A1 | XYHYC185246 | MG600076 |
| Tropivirus | Tropivirus awaski | tropivirus A1 | ZGLXR119682 | MG600091 |
|  | Tropivirus betero | tropivirus B1 | LPWC175499 | MG600083 |
