# Supplementary material for: Genomic characterization of a novel sakobuvirus (family Picornaviridae) from a European badger (Meles meles) in Hungary
Source: Arch Virol. 2025 Feb 20;170(3):63. doi: 10.1007/s00705-025-06234-4 (PMC11842475; doi:10.1007/s00705-025-06234-4)
Supplement: Supplementary file 4 — Supplementary Material 4 [file 705_2025_6234_MOESM4_ESM.docx]

**Genomic characterization of a novel sakobuvirus (family *Picornaviridae*) from a European badger (*Meles meles*) in Hungary**

Supplementary file

**Supplementary Fig. S1.: Methods and configuration used for bioinformatic analysis.** Faecal samples from B9, B10 and B40 animals were randomly selected for viral metagenomics and next-generation sequencing (VM-NGS) [1,2] and the data were analysed by DIAMOND/MEGAN6 method [3]. Diamond (ver. 2.1.9) setup: command: diamond blastx --db viral.1.protein_diamond_db.dmnd --query UPL21-001344_R1R2.fastq --out UPL21-001344_R1R2_vs_viral.1.protein_diamond_db.daa --evalue 0.001 --max-target-seqs 5 --id 50 --query-cover 50 --outfmt 100 --threads 14 --tmpdir F:\TEMP; Source file: UPL21-001344_R1 / UPL21-001344_R2 fastq files, these two files contain 47,851,176 reads; MEGAN Community Edition (version 6.25.10, built 27 Jun 2024) setup: Reads=415,199 Assigned=207,888 (readCount) MinScore=50.0 MaxExpected=0.01 TopPercent=10.0 MinSupportPercent=0.01 MinSupport=41 disabledTaxa=13 LCA=naive mode=BlastX; For the MEGAN6 software package, we used a custom-made taxonomic database file based on the nr database file, accessed 28.03.2023: protein_taxonomy_map20230328.db


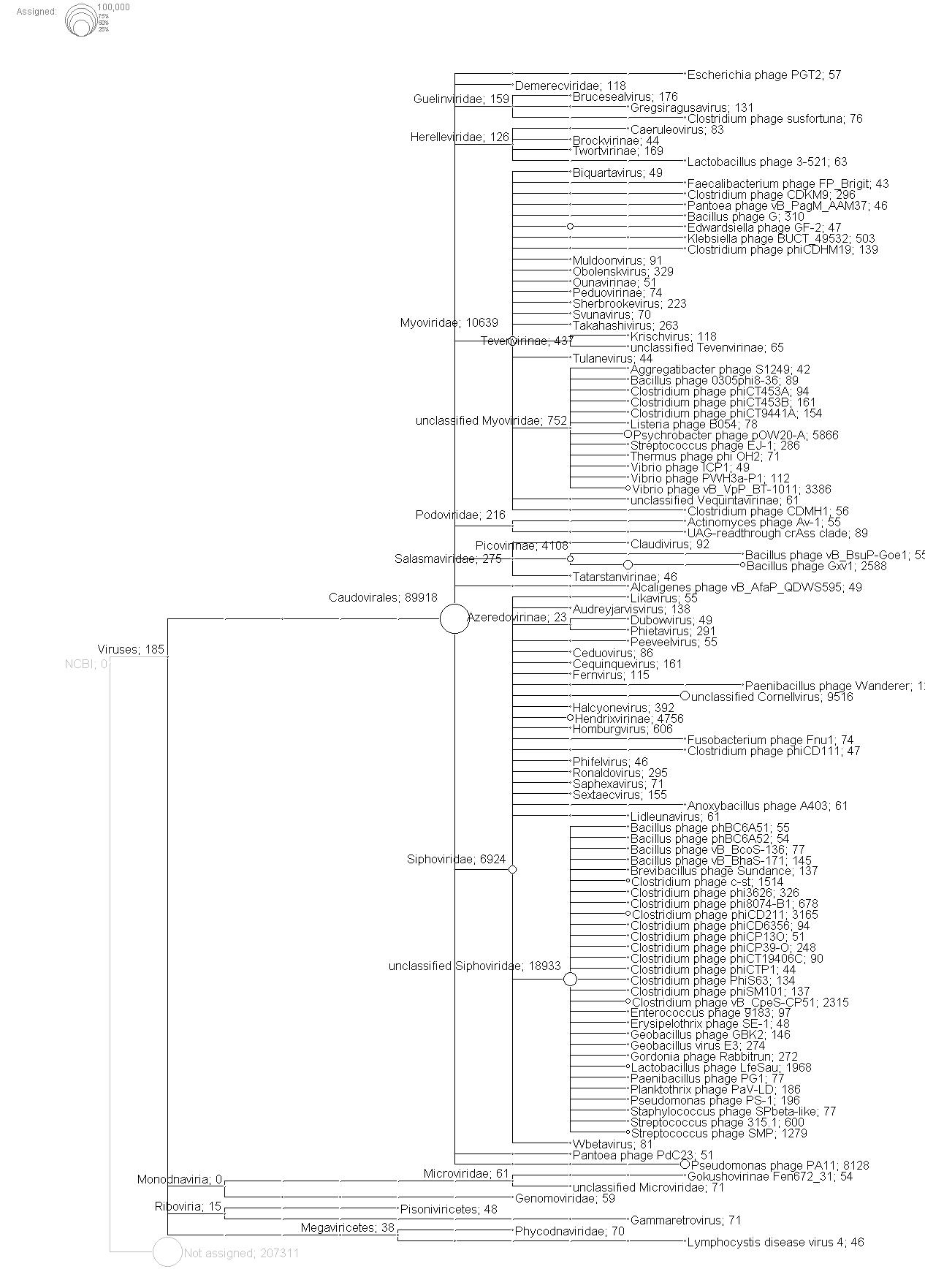


**References**

1. Boros Á, Albert M, Urbán P et al (2022) Unusual “Asian-origin” 2c to 2b point mutant canine parvovirus (*Parvoviridae*) and canine astrovirus (*Astroviridae*) co-infection detected in vaccinated dogs with an outbreak of severe haemorrhagic gastroenteritis with high mortality rate in Hungary. Vet. Res Comm. 46(4), 1355-1361. <https://doi.org/10.1007/s11259-022-09997-2>
2. Boros Á, Pankovics P, László Z et al (2023) The genomic and epidemiological investigations of enteric viruses of domestic caprine (Capra hircus) revealed the presence of multiple novel viruses related to known strains of humans and ruminant livestock species. Microbiology Spectrum, 11(6), e02533-23. <https://doi.org/10.1128/spectrum.02533-23>
3. Bağcı C, Patz S, Huson DH (2021) DIAMOND+MEGAN: Fast and Easy Taxonomic and Functional Analysis of Short and Long Microbiome Sequences. Curr Protoc 1(3):e59. <https://doi.org/10.1002/cpz1.59>. Erratum in: Curr Protoc 2(8):e552. <https://doi.org/10.1002/cpz1.552> Erratum in: Curr Protoc 2(8):e551. <https://doi.org/10.1002/cpz1.551>
