# Supplementary material for: Genomic characterization of a novel sakobuvirus (family Picornaviridae) from a European badger (Meles meles) in Hungary
Source: Arch Virol. 2025 Feb 20;170(3):63. doi: 10.1007/s00705-025-06234-4 (PMC11842475; doi:10.1007/s00705-025-06234-4)

**Genomic characterization of a novel sakobuvirus (family *Picornaviridae*) from a European badger (*Meles meles*) in Hungary**

Supplementary file

**Supplementary Fig. S2.: Alignment of sakobuvirus 5’UTR/IRES nucleotide sequences**


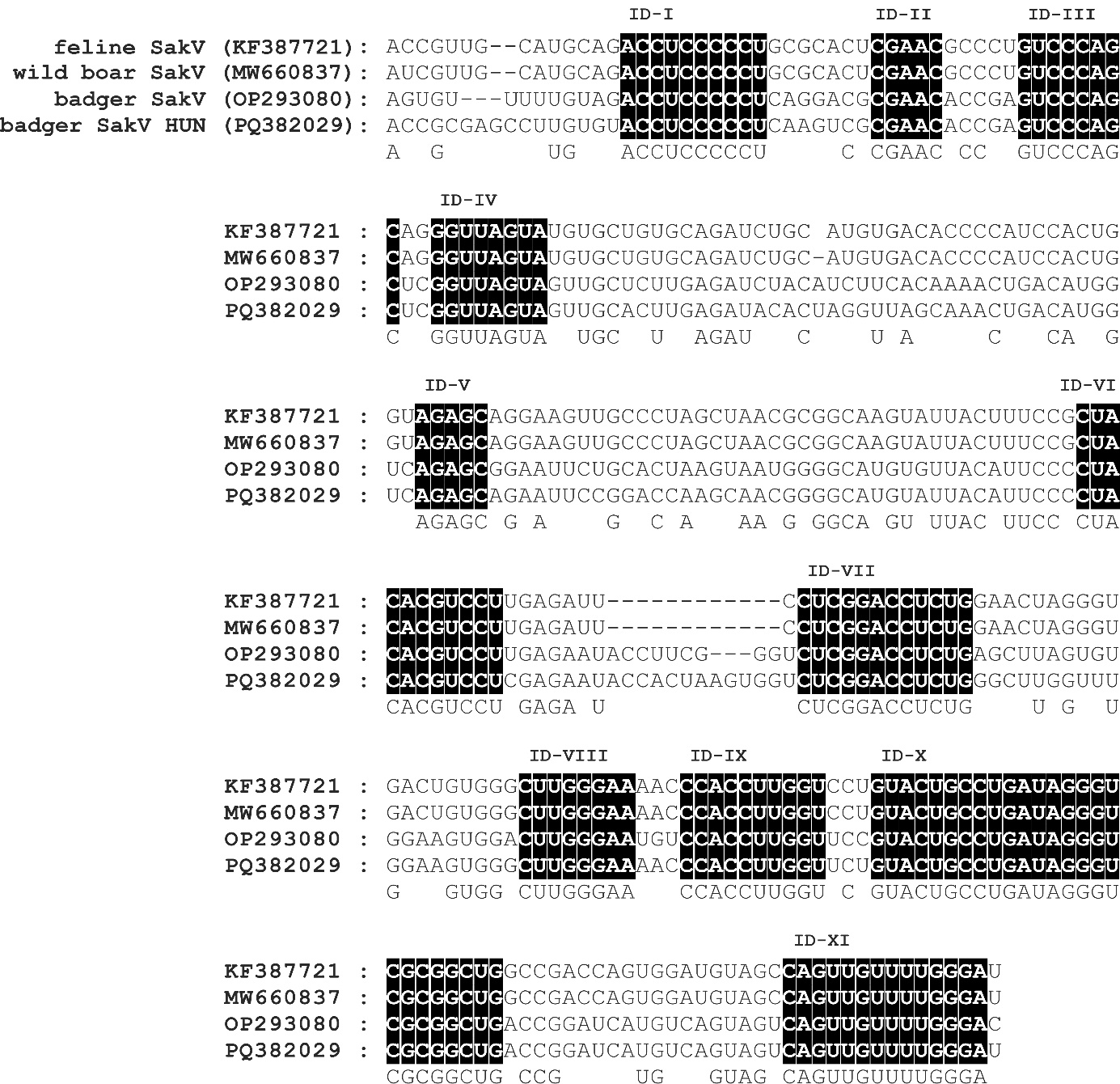

Supplement: Supplementary file 5 — Supplementary Material 5 [file 705_2025_6234_MOESM5_ESM.docx]
