# Supplementary material for: Genomic characterization of a novel sakobuvirus (family Picornaviridae) from a European badger (Meles meles) in Hungary
Source: Arch Virol. 2025 Feb 20;170(3):63. doi: 10.1007/s00705-025-06234-4 (PMC11842475; doi:10.1007/s00705-025-06234-4)

**Genomic characterization of a novel sakobuvirus (family *Picornaviridae*) from a European badger (*Meles meles*) in Hungary**

Supplementary file

**Supplementary Fig. S4**.: **The RNA secondary structure prediction for the 5′ and 3′ untranslated regions (UTRs) of badger sakobuvirus (BaSakV) strain SakV/badger/B40B/2022/HUN (PQ382029).**

**A)** The secondary RNA structure of the 5’UTR is based on the ClustalW alignment of the 5′UTR/IRES nucleotide sequences of feline (KF387721), wild boar (MW660837), and badger (OP293080) sakobuviruses available in GenBank. The type IV internal ribosome entry site (IRES) of BaSakV has been annotated based on picornavirus conventions. The domains are labelled as II and III, while the helical segments are marked as II1, II2, III1, III2, etc., and hairpins as IIIa, IIIb, etc., to keep the nomenclature consistent. Conserved domains and the AUG start codon of the polyprotein are indicated by shaded boxes. The nucleotide sequences marked by the dashed line is identical in the reference species Sakobuvirus aportufeli isolate FFUP1 (KF387721), wild boar sakobuvirus isolate WBSA (MW660837), and novel species of badger sakobuviruses strains SaKoV/Badger/3A_2019/ITA (OP293080) and SakV/badger/B40B/2022/HUN (PQ382029) viruses. The conserved nucleotide motifs in hairpins IIId (UUGGGAAA, positions 612-619) and IIIe (GCCUGAUAGGGU, positions 640-651) are shared across SakV sequences.

**B)** The secondary RNA structure of the 3’UTR of BaSakV is based on manual alignment of corresponding sequences of the aforementioned sakobuvirus sequences. The figure is consisting of a detailed depiction of the 'barbell-like' structure, along with the characteristic poly(Y) tract linked to this formation, highlighting conserved nucleotides shared with other picornavirus species. Based on the sequence alignment, the "barbell”-like secondary RNA structure with its structural element (GAUAUAAAGACCC, GACCCUAA) and p(Y) tract at the apical tip (UUUUCCG) was present in the study strain.


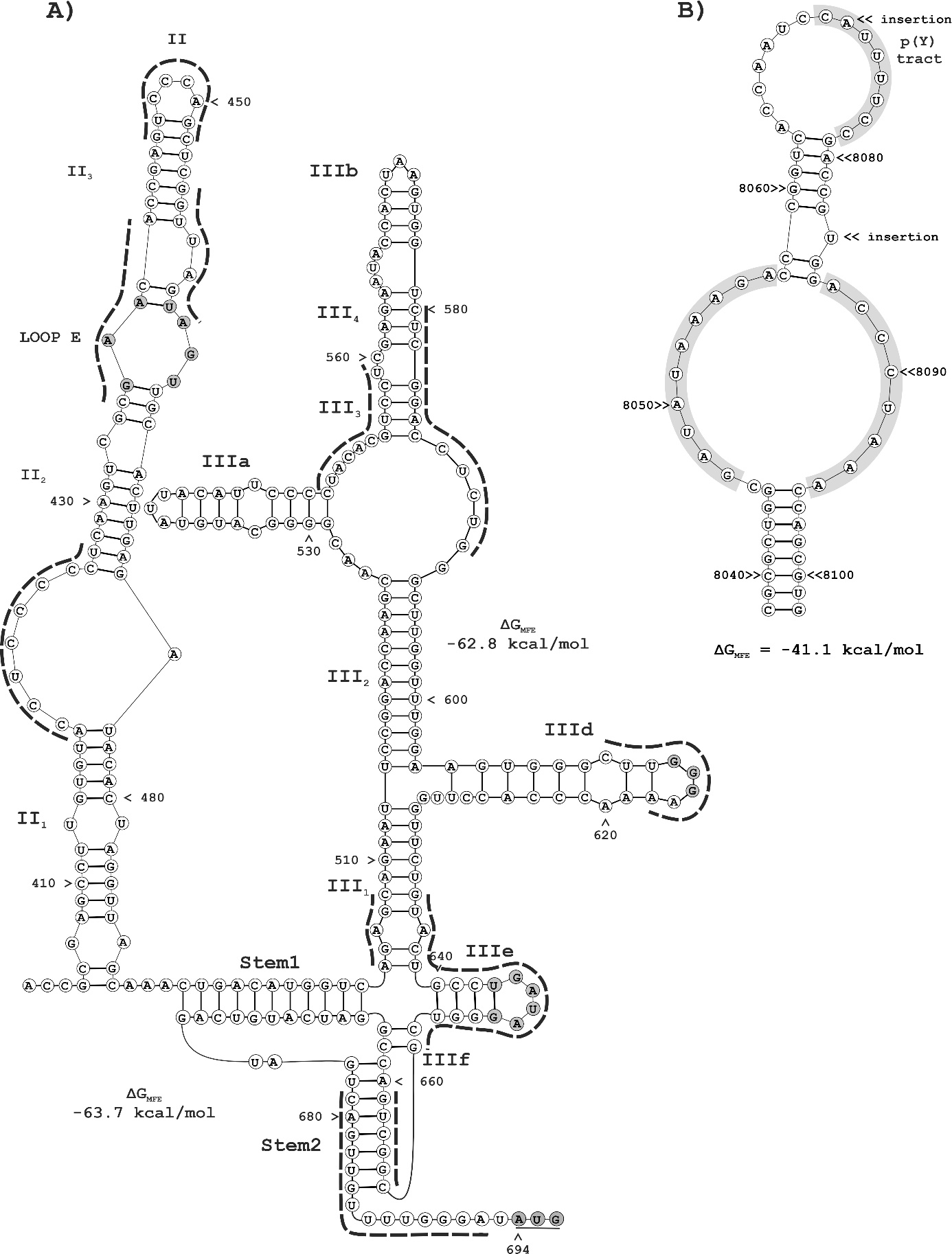

Supplement: Supplementary file 7 — Supplementary Material 7 [file 705_2025_6234_MOESM7_ESM.docx]
