# Supplementary material for: Genomic characterization of a novel sakobuvirus (family Picornaviridae) from a European badger (Meles meles) in Hungary
Source: Arch Virol. 2025 Feb 20;170(3):63. doi: 10.1007/s00705-025-06234-4 (PMC11842475; doi:10.1007/s00705-025-06234-4)

**Genomic characterization of a novel sakobuvirus (family *Picornaviridae*) from a European badger (*Meles meles*) in Hungary**

Supplementary file

**Supplementary Fig. S5.: Standard protein BLAST between badger sakobuvirus (PQ382029) and unclassified sakobuvirus sequences**

The BLASTp alignment of the entire protein sequence of the Query: badger sakobuvirus strain SakV/badger/B40B/2022/HUN (PQ382029) compared to unclassified Sakobuvirus (taxid:1659782) sequences.


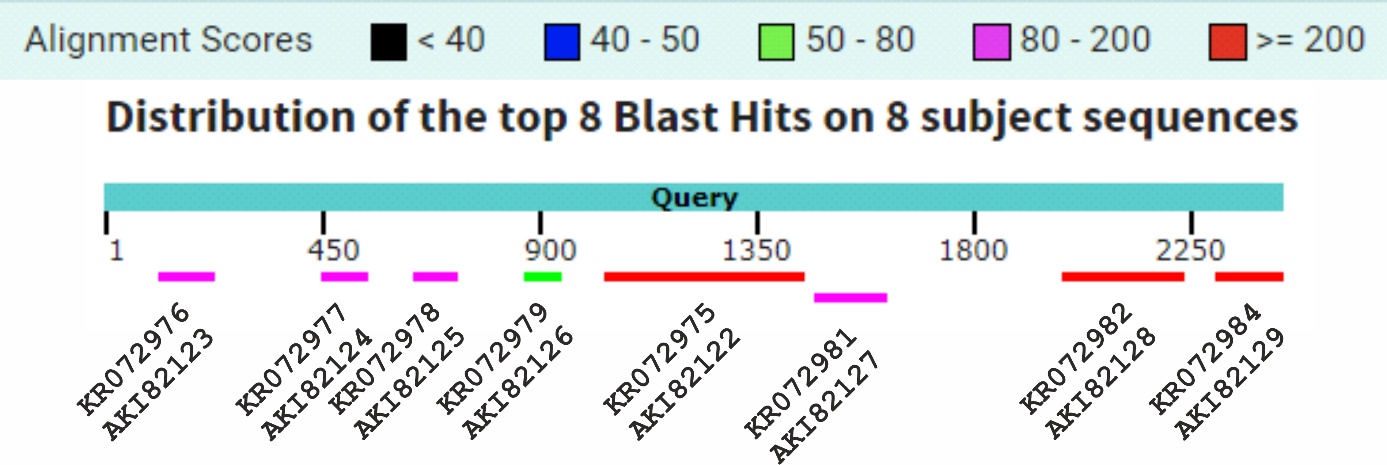


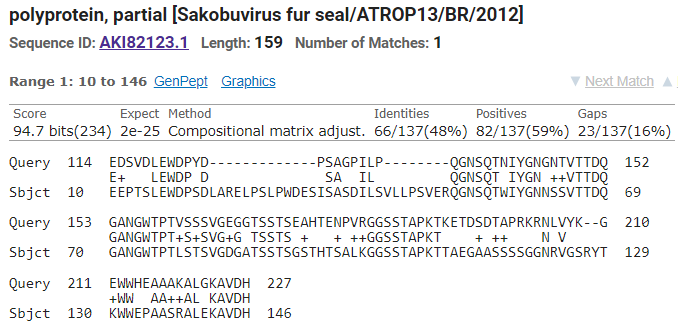


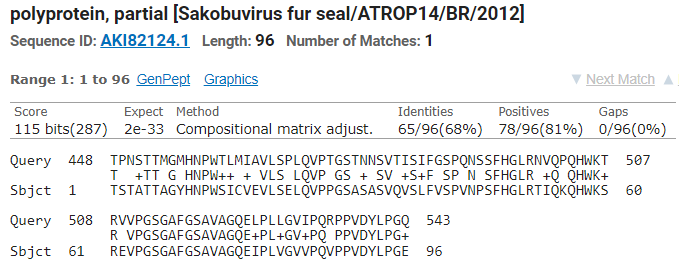


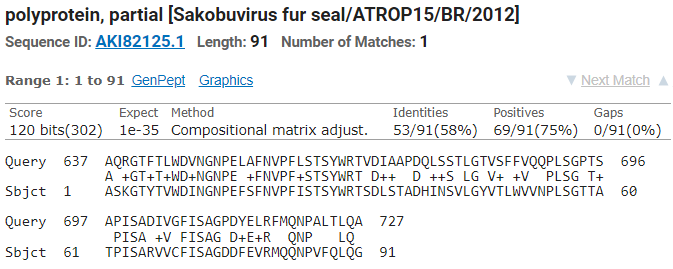


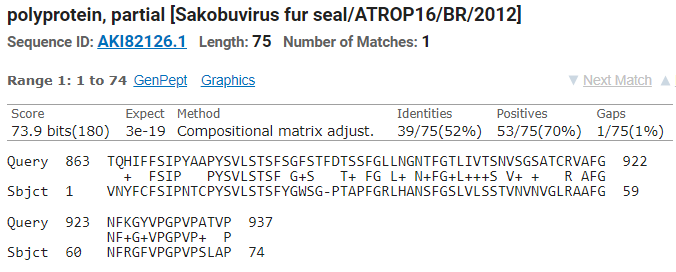


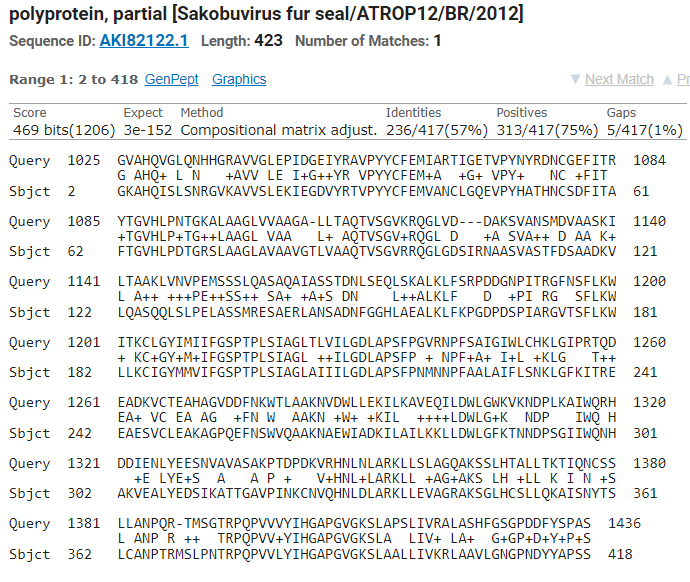


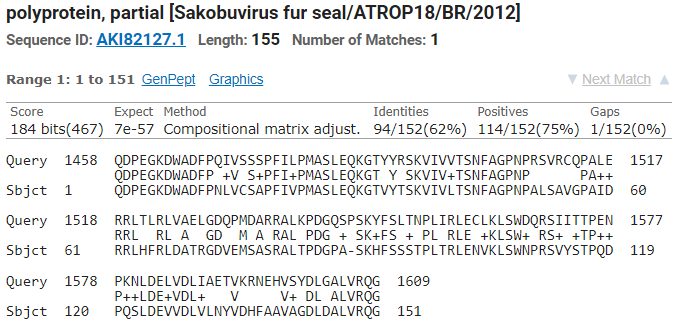


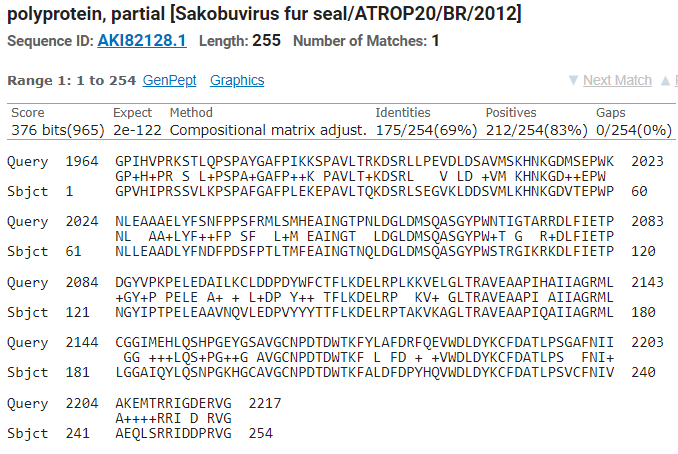


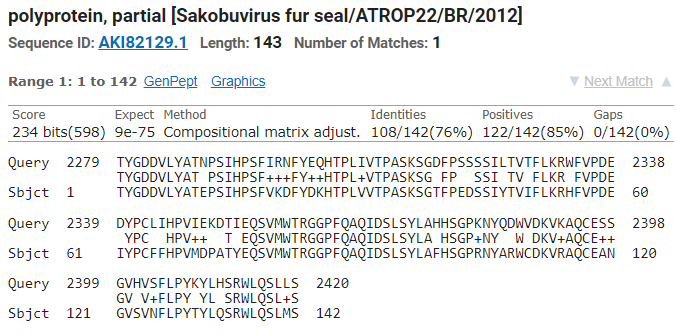

Supplement: Supplementary file 8 — Supplementary Material 8 [file 705_2025_6234_MOESM8_ESM.docx]
