# Supplementary material for: Genomic characterization of a novel sakobuvirus (family Picornaviridae) from a European badger (Meles meles) in Hungary
Source: Arch Virol. 2025 Feb 20;170(3):63. doi: 10.1007/s00705-025-06234-4 (PMC11842475; doi:10.1007/s00705-025-06234-4)
Supplement: Supplementary file 9 — Supplementary Material 9 [file 705_2025_6234_MOESM9_ESM.docx]

**Genomic characterization of a novel sakobuvirus (family *Picornaviridae*) from a European badger (*Meles meles*) in Hungary**

Supplementary file

**Supplementary Methods: Detailed description of the nucleic acid extraction, complete genome acquisition, sequencing, and phylogeny used in the manuscript.**

The complete viral genome of strain SakV/badger/B40B/2022/HUN was obtained using total RNA extracted with TRIzol Reagent (Thermo Fisher Scientific, USA) and identified by primer-walking [1], 5’/3’RACE [2] and Template Switching Oligonucleotide (TSO) methods [3,4], directly sequenced using BigDye Terminator v.1.1 Cycle Sequencing Kit, and then run on an automated sequencer (AB3500 Genetic Analyzer, Applied Biosystems, Japan). The obtained sequence data were analysed using the GenDoc (v. 2.7), EMBL-EBI [5], MUSCLE [6], MEGA11 [7], and SimPlot (v. 3.5.1) software packages. Comparative genome and phylogenetic diagrams were further refined and post-edited using the CorelDRAW v. 24.1.0.360) software.

**Total RNA extraction**: The initial step in extracting total RNA from faecal samples involved diluting the faecal specimens with a minimum of 1 mL of phosphate-buffered saline (PBS) solution. Following thorough homogenization, the samples were treated with equal volume of Genetron 113 (1,1,2-Trichloro-1,2,2-trifluoroethane; Sigma Aldrich, USA). The resulting Genetron-treated faecal suspensions were centrifuged at X g for 5 minutes at 4°C and from the obtained supernatant, 150 µL was carefully collected for RNA extraction. The extraction was performed using TRIzol^®^ Reagent (Thermo Fisher Scientific, USA) in strict accordance with the manufacturer's protocol. Finally, the total RNA was resuspended in 20 µL of nuclease-free water (NFW, AccuGene, Lonza, USA) to ensure the integrity of the extract.

**Template Switching Oligonucleotide (TSO) method**: For the reverse transcription reaction, Template Switching RT Enzyme Mix (M0466S, New England Biolabs, Ipswich, MA, USA) sequence specific reverse primers (stock concentration 10 µM) and a template-switching oligonucleotide (TSO-RT): 5’-GCTAATCATTGCAAGCAGTGGTATCAACGCAGAGTACATrGrGrG-3’ (r: ribose) (stock concentration 75 µM) was used according to the manufacturer's instructions. In the PCR reaction mixture with a final volume of 25µl, a total of 18.75µl of NFW, 0.25µl of DreamTaq Enzyme (5U/µl, Thermo Fisher), 2.5µl of 10x DreamTaq buffer (Thermo Fisher), 0.5µl of 10M dNTP mix (Promega, Madison, USA), 0.5µl of 20µM TSO-Adapter (5’-CATTGCAAGCAGTGGTATCAAC-3’) and sequence-specific (20 µM) primers were used. Finally, 2µl of cDNA product was added to the PCR mix. All specimens were re-tested by the conventional RT-PCR method using Sakobuvirus-3D-SCR-F and modified 3’ RACE primers.

**Description of the sakobuvirus screening method**: All faecal specimens (N=13) and available tissue samples (N=12) from European badgers were re-tested by conventional RT-PCR method using Sakobuvirus-3D-SCR-F (5’-TCNNZNCACTTTGCTGGTGT-3’, N: any of four nucleotides) screening primer and a modified 3’ RACE method. Briefly summarizing the method, we designed an anchored oligo dT-Adapter oligonucleotide primer, OligodT-anchorNEW (OTAN: 5’-AAAGGTCTAGAGGTGATCAGTTAACTAGTTAGTACTCGAGGGTTTTTTTTTTTTTTTV-3’) targeting the poly(A) sequence at the 3’end of the viral genome. For cDNA synthesis, a denaturing mix was prepared in a final volume of 10µl, which contained 4µl of NFW, 5µl of total RNA extract and 1µl of 20 µM OTAN primer. The prepared mix was incubated for 5 minutes at 65ºC, then a total volume of 10µl of RT mix was added to the denaturing mix, which consists of 3.5µl of NFW, 4µl of 5x Maxima H minus RT buffer (Thermo Fisher), 1µl of 10mM dNTP mix, 0.5 µl of 40U/µl RiboLock Ribonuclease Inhibitor (Thermo Fisher) and 1µl of 200U/µl Maxima H minus RT enzyme (Thermo Fisher). The reverse transcription reaction was conducted on 50ºC for 55 minutes, then the RT enzyme was inactivated by incubation at 85ºC for 5 minutes. The cDNA was treated with 0.5µl 5U/µl RNase H (Thermo Fisher) enzyme at 37ºC for 20min, then 65ºC for 10min and stored at -20ºC until further use. In the first PCR reaction, 1 µl of 20 µM specific forward Sakobuvirus-3D-SCR-F primer and 1 µl of 20 µM ADAPTER_B (5'-AAA GGT CTA GAG GTG ATC AGT TAA CTA GTT AGT ACT CGA GGG-3') reverse primer were used. while for the second PCR reaction an inner, hemi-nested ADAPTER_B-nestD (5'-TCA GT TAAC TAG TTA GTA CTC GAG GG-3') reverse primer was applied.

**Direct sequencing method**: PCR products were directly sequenced by the Sanger method using BigDye Terminator v.1.1 Cycle Sequencing Kit (Thermo Scientific, USA) according to the manufacturer’s instruction and then run on an automated sequencer AB3500 Genetic Analyzer (Applied Biosystems, Japan).

**Phylogenetic method**: For the phylogenetic analyses, the Kalign algorithm service hosted at the EMBL-EBI (<https://www.ebi.ac.uk/jdispatcher>) framework [5], MUSCLE [6] and the MEGA11 [7] were used and maximum likelihood based phylogenetic analysis was done using LG+G+(F) substitutional models with MEGA11 [7]. Bootstrap values were determined with 1000 replicates and the tree was drawn to scale, with branch lengths representing the number of substitutions per site.

**References**

1. Sverdlov E, Azhikina T (2005) Primer Walking. In: John Wiley & Sons Ltd, Chichester. <http://www.els.net>, <http://doi.org/10.1038/npg.els.0005382>
2. Boros Á, Pankovics P, Simmonds P et al (2011) Novel positive-sense, single-stranded RNA (+ssRNA) virus with dicistronic genome from intestinal content of freshwater carp (*Cyprinus carpio*). PLoS One, e29145. <https://doi.org/10.1371/journal.pone.0029145>
3. Adamopoulos PG, Tsiakanikas P, Stolidi I et al (2022) A versatile 5' RACE-Seq methodology for the accurate identification of the 5' termini of mRNAs. BMC Genomics 23(1):163. <https://doi.org/10.1186/s12864-022-08386-y>
4. Klink P, Harms D, Altmann B et al (2023) Molecular characterisation of a rabbit Hepatitis E Virus strain detected in a chronically HEV-infected individual from Germany. One Health 16:100528. <https://doi.org/10.1016/j.onehlt.2023.100528>
5. Madeira F, Madhusoodanan N, Lee J et al (2024) The EMBL-EBI Job Dispatcher sequence analysis tools framework in 2024. Nucl Acid Res 52(W1):W521-W525. <https://doi.org/10.1093/nar/gkae241>
6. Edgar RC.(2004) MUSCLE: multiple sequence alignment with high accuracy and high throughput. Nucleic Acids Res 32(5):1792-7. <https://doi.org/10.1093/nar/gkh340>
7. Tamura K, Stecher G, Kumar S. (2021) MEGA11: Molecular Evolutionary Genetics Analysis Version 11. Mol Biol Evol 38(7):3022-3027. <https://doi.org/10.1093/molbev/msab120>
